# Veliparib Is an Effective Radiosensitizing Agent in a Preclinical Model of Medulloblastoma

**DOI:** 10.3389/fmolb.2021.633344

**Published:** 2021-04-29

**Authors:** Jessica Buck, Patrick J. C. Dyer, Hilary Hii, Brooke Carline, Mani Kuchibhotla, Jacob Byrne, Meegan Howlett, Jacqueline Whitehouse, Martin A. Ebert, Kerrie L. McDonald, Nicholas G. Gottardo, Raelene Endersby

**Affiliations:** ^1^Brain Tumour Research Program, Telethon Kids Cancer Centre, Telethon Kids Institute, Perth, WA, Australia; ^2^Centre for Child Health Research, University of Western Australia, Perth, WA, Australia; ^3^School of Physics, Mathematics and Computing, University of Western Australia, Perth, WA, Australia; ^4^Radiation Oncology, Sir Charles Gairdner Hospital, Perth, WA, Australia; ^5^Brain Cancer Consultancy, Sydney, NSW, Australia; ^6^Department of Paediatric Oncology and Haematology, Perth Children’s Hospital, Perth, WA, Australia

**Keywords:** Medulloblastoma, radiotherapy, veliparib, DNA repair, poly(ADP-ribose) polymerase

## Abstract

Medulloblastoma is the most common malignant childhood brain tumor, and 5-year overall survival rates are as low as 40% depending on molecular subtype, with new therapies critically important. As radiotherapy and chemotherapy act through the induction of DNA damage, the sensitization of cancer cells through the inhibition of DNA damage repair pathways is a potential therapeutic strategy. The poly-(ADP-ribose) polymerase (PARP) inhibitor veliparib was assessed for its ability to augment the cellular response to radiation-induced DNA damage in human medulloblastoma cells. DNA repair following irradiation was assessed using the alkaline comet assay, with veliparib inhibiting the rate of DNA repair. Veliparib treatment also increased the number of γH2AX foci in cells treated with radiation, and analysis of downstream pathways indicated persistent activation of the DNA damage response pathway. Clonogenicity assays demonstrated that veliparib effectively inhibited the colony-forming capacity of medulloblastoma cells, both as a single agent and in combination with irradiation. These data were then validated *in vivo* using an orthotopic implant model of medulloblastoma. Mice harboring intracranial D425 medulloblastoma xenografts were treated with vehicle, veliparib, 18 Gy multifractionated craniospinal irradiation (CSI), or veliparib combined with 18 Gy CSI. Animals treated with combination therapy exhibited reduced tumor growth rates concomitant with increased intra-tumoral apoptosis observed by immunohistochemistry. Kaplan–Meier analyses revealed a statistically significant increase in survival with combination therapy compared to CSI alone. In summary, PARP inhibition enhanced radiation-induced cytotoxicity of medulloblastoma cells; thus, veliparib or other brain-penetrant PARP inhibitors are potential radiosensitizing agents for the treatment of medulloblastoma.

## Introduction

Brain tumors represent one of the leading causes of mortality in children, with medulloblastoma the most common childhood brain cancer (Millard and De Braganca, 2016). Medulloblastoma is a heterogeneous group of cancers that can been divided into four core molecular subgroups: SHH, WNT, Group 3, and Group 4 (Thompson et al., 2006; Louis et al., 2016). These subgroups can be further classified into 13 subtypes based on genomic, epigenomic, proteomic, and clinical features (Hovestadt et al., 2020). Five-year overall survival rates for medulloblastoma range from 40 to 98%, depending on molecular subtype (Hovestadt et al., 2020), with *MYC*-amplified Group 3 medulloblastoma associated with very poor survival rates. Despite significant progress in our understanding of the underlying molecular drivers of these tumors, this has not yet been translated into improved outcomes.

The standard treatment regimen for medulloblastoma consists of maximal safe tumor resection followed by craniospinal irradiation (CSI) and multi-agent chemotherapy (Martin et al., 2014; Northcott et al., 2019). As radiotherapy and chemotherapy induce DNA damage, sensitizing medulloblastoma cells to these treatments through inhibiting DNA repair pathways is a potential therapeutic strategy (Carrassa and Damia, 2017). We recently performed a drug screen that identified kinase inhibitors of the DNA damage response (DDR) pathway and cell cycle machinery as potent agents against *MYC*-amplified Group 3 medulloblastoma in combination with chemotherapy (Endersby et al., 2021). The DDR is a carefully orchestrated network which allows cells to sense problems in their DNA, arrest cell cycle progression and repair DNA damage. The poly-(ADP-ribose) polymerase (PARP) family of proteins are important facilitators of DNA damage repair. While there are 18 members of the PARP family, PARP1 and PARP2 are the most important for DNA damage repair (Kamaletdinova et al., 2019). PARPs bind to DNA at various sites of damage which stimulates them to synthesize PAR chains (PARylation). These PAR chains act as docking sites for DNA repair proteins, facilitating their recruitment to sites of DNA damage (Pommier et al., 2016). A number of PARP inhibitors have been developed and may be used clinically to treat cancers with homologous recombination (HR) deficiency, such as those with mutations in *BRCA1*, *BRCA2*, and *PALB2*. In these cancers, PARP inhibitors can induce synthetic lethality, and this approach has had some success particularly in breast and ovarian cancer (Janysek et al., 2021). This synthetic lethality approach is unlikely to be widely successful in medulloblastoma, as very few patients harbor mutations in genes encoding HR machinery. However, PARP inhibitors have recently been investigated for their ability to sensitize brain cancer cells to chemotherapy and radiotherapy (Van Vuurden et al., 2011; Chornenkyy et al., 2015; Jue et al., 2017) and it is this approach that we sought to test in medulloblastoma.

There is evidence that PARP inhibition can sensitize cancer cell lines to radiation, including cells derived from pediatric brain tumors. PARP inhibition increased DNA damage, and reduced cell viability and proliferation when combined with irradiation in pediatric high-grade glioma, ependymoma, and diffuse intrinsic pontine glioma (DIPG) cell lines *in vitro* (Van Vuurden et al., 2011; Chornenkyy et al., 2015). Combined PARP inhibition and radiotherapy has also increased survival in mouse models of high-grade astrocytoma (Chornenkyy et al., 2015). In medulloblastoma, it has been demonstrated that PARP inhibition has the potential to sensitize cells to chemotherapy *in vivo* (Daniel et al., 2010). Radiosensitization of medulloblastoma cells has been demonstrated *in vitro* (Van Vuurden et al., 2011), however this has not yet been tested *in vivo* in orthotopic xenograft models.

Of the PARP inhibitors developed so far, veliparib is the most clinically advanced in children. A phase I trial has examined veliparib in combination with temozolomide for the treatment of recurrent CNS tumors in children, including two patients diagnosed with medulloblastoma (Su et al., 2014); where veliparib was well tolerated and stable disease was observed in four patients. A recent phase I/II clinical trial examined the use of veliparib in combination with temozolomide and radiotherapy for treatment of DIPG (Baxter et al., 2020). While veliparib was well-tolerated, no survival benefit was shown at interim analysis, and the trial was stopped. A further phase I/II clinical trial examining the use of veliparib in combination with temozolomide and radiotherapy for treatment of pediatric glioma is currently underway (clinicaltrials.gov ID#NCT03581292). Recently, veliparib in combination with radiation was shown to significantly increase survival of glioblastoma patient-derived xenograft (PDX) mouse models (Jue et al., 2017). Therefore, we sought to investigate methods that may improve treatment outcomes for high-risk medulloblastoma patients by preclinically evaluating the radiosensitizing potential of PARP inhibition in *MYC-*amplified Group 3 medulloblastoma models. Despite promising data from PARP inhibitors like niraparib and olaparib (Murai et al., 2012), given that veliparib is the most clinically tested PARP inhibitor for children with brain cancer, we investigated a potential role for veliparib in medulloblastoma to ensure rapid clinical translatability.

## Methods

### Cell Culture

D425 and D283 human medulloblastoma cells were a gift from Prof. Darell Bigner of Duke University, United States (Friedman et al., 1985; Bigner et al., 1990). STR analysis and sequencing of previously reported genetic alterations confirmed the identity of all cell lines. All cultures were incubated at 37°C with 5% CO_2_ and confirmed mycoplasma-free using a MycoAlert^TM^ Mycoplasma Detection Kit (Lonza). D425 cells were cultured in modified IMEM (#A10489-01, Gibco) supplemented with GlutaMAX (#35050-061, Invitrogen), 10% fetal bovine serum (FBS, Cell Sera), and 10 μM HEPES (#15630-080, Gibco). D425 cells were transduced with pCL20-MSCV-GFP-ires-Luc2 lentiviral particles to drive expression of GFP and luciferase (referred to as D425GiL). D283 cells were cultured in MEMα (#12561072, Gibco) supplemented with GlutaMAX (Invitrogen) and 10% FBS. D283 were transduced with MSCV-ires-pacLuc2 retroviral particles to drive expression of a puromycin acetyltransferase and codon-optimized firefly luciferase fusion protein (referred to as D283Luc2). Viral constructs and packaging plasmids were kindly provided by Drs. Richard Williams and Arthur Nienhuis of St. Jude Children’s Research Hospital, United States.

### In vitro Dose Response Assay

Veliparib (MedChem Express) was dissolved in DMSO (10 mM). D425Gil or D283Luc2 cells (5,000/well) were seeded into black-walled 384-well plates (Costar) and compounds were applied using a D300e digital dispenser (Tecan). Viability was assessed after 72 h using alamarBlue [0.6 mM resazurin, 1 mM potassium hexacyanoferrate (II) trihydrate, 1 mM potassium hexacyanoferrate (III), 2.5% methylene blue]. Resorufin fluorescence (excitation 570 nm, emission 590 nm) was used to calculate the percentage of viable cells relative to control (DMSO) wells. The effective dose that inhibits 50% of cells (ED50) was determined using data pooled from at least three independent experiments. For drug-radiation interaction assays, cells were treated with increasing concentrations of veliparib alone, X-ray radiation alone [1, 2.5, 5, or 7.5 Gy, delivered using an XRAD SmART 225-cx (Precision X-ray) (Supplementary Methods)], or the combination of both veliparib and radiation. The combinatorial treatment effects were assessed using Combenefit software (Di Veroli et al., 2016). At least three independent experiments were performed.

### Comet Assays

D425Gil cells were treated with DMSO (0.1%) or veliparib (10 μM). After the addition of drug, cells were immediately placed on ice, then exposed to 10 Gy γ-radiation using a cesium source irradiator (Gammacell 3000, MDS Nordion) and returned to ice. Cells were either immediately resuspended in 1% low melting point agarose and spread onto a glass slide, or incubated at 37°C in 5% CO_2_ for 10, 20, 40, 60, 80, 100, or 120 min prior to processing. Slides were immersed in lysis solution (2.5 M NaCl, 0.1 M EDTA, 10 mM Tris–HCl and 1% Triton X-100) at 4°C for 1 h, then washed twice in alkaline solution (0.3 M NaOH, 1 mM EDTA). DNA was separated via electrophoresis in alkaline solution for 20 min at 18 V, 4°C. A reference standard slide with pre-irradiated (10 Gy) cells was included in every electrophoresis run for standardization. Slides were washed for 10 min and propidium iodide added for visualization. OpenComet software (Gyori et al., 2014) was used to determine the proportion of damaged DNA in each cell by calculation of percentage tail DNA. A minimum of three experimental replicates were performed.

### Immunofluorescence

D425 and D283 cells were seeded onto Matrigel (BD Biosciences) coated coverslips and then treated with DMSO (0.1%) or 10 μM veliparib, followed by either 0 or 2 Gy γ-radiation (Gammacell 3000, MDS Nordion). Cells were fixed 24 h post-irradiation and stained using the following antibodies: γH2AX [Cell Signaling Technologies (CST), #9718S, 1:400], RPA32/RPA2 (CST #2208S, 1:200), AlexaFluor488 anti-rabbit (Life Technologies, #A11008, 1:400) and AlexaFluor568 anti-rat (Life Technologies, #A11077, 1:200). Nuclei were stained using NucBlue (Life Technologies, #R37605), and coverslips mounted in VectorShield (Vector Labs). Images were taken using a Nikon Ti-E microscope and images analyzed using NIS Elements software (Nikon).

### Flow Cytometry

Cell cycle distribution was analyzed using EdU (added 45 min before harvest) to label cells in S phase and DAPI to label DNA content. Cells were treated and harvested as indicated in the figure legends. Cells were stained using the Click-iT EdU AlexaFluor488 kit (Invitrogen). Samples were analyzed using an LSRFortessa X20 (BD) and results were visualized and quantified using FlowJo software as previously described (Andradas et al., 2021). Data are pooled from two independent experiments and show the mean with standard deviation (SD).

### Protein Analysis by Immunoblotting

D425GiL and D283Luc2 cells were treated with DMSO (0.1%) or 10 μM veliparib, alone or combined with 10 Gy γ-radiation (Gammacell 3000, Nordion). Cells were lysed after 24 h with radioimmunoprecipitation assay (RIPA) buffer containing protease and phosphatase inhibitors (Roche). Protein (30 μg/lane) was separated using 4–12% NuPAGE Bis-Tris gels (Invitrogen) and transferred to nitrocellulose membranes. Membranes were immunoblotted with primary antibodies and horseradish peroxidase-conjugated secondary antibodies (1:5000, Cytiva) which were detected using Supersignal West Dura (Pierce) or Clarity Western ECL (Bio-Rad) and images collected using a ChemiDoc (Bio-Rad). Primary antibodies used were phosphorylated (p-) CHK1^*Ser*345^ (#2348), p-CHK2^*Thr*68^ (#2661), p53 (#9282), PAR (#83732), PARP (#9542), CHK1 (#2360), CHK2 (#6334), and γH2AX (#9718S) from CST, and β-actin (Sigma-Aldrich, #A1978). Data are representative of two independent experiments.

### Colony-Forming Assays

Medulloblastoma cells were treated with DMSO (0.1%) or veliparib (10 μM), then either untreated or exposed to 2 Gy γ-radiation (Gammacell 3000, Nordion) prior to suspending in media containing 1.25% methylcellulose (STEMCELL Technologies) and plating. After 14 days, colonies at least 100 μm in diameter were counted. Three independent experiments were performed.

### Orthotopic Xenograft Model of Medulloblastoma

D425Gil cells (5 × 10^5^) suspended in Matrigel (BD Biosciences) were implanted into the right cerebral cortex of 7–10 weeks old NOD/Rag1^–/–^ mice (Jackson Laboratories). Tumor growth was monitored weekly using bioluminescence imaging (BLI, IVIS Spectrum, Caliper). Mice were randomized into treatment groups with equivalent mean bioluminescence (photons per second per centimeter squared per steradian, abbreviated as p/s). Veliparib dissolved in 20% Captisol^®^ was delivered *per os* (p.o.) twice daily (12.5 mg/kg/dose). When delivered in combination, veliparib was administered 1 h prior to radiotherapy. Radiotherapy was delivered using an X-RAD SmART (Precision X-ray) employing cone-beam CT guidance with fully assessed spatial and dosimetric accuracy (Feddersen et al., 2019; Supplementary Material). For CSI, mice were anesthetized using isoflurane, and 18 Gy was delivered as nine 2 Gy fractions on sequential weekdays. Three sets of two lateral coplanar beams with 40 mm square collimation were delivered to three separate isocentres, with the first set of beams targeting the brain and cervical spine, the second targeting the thoracic spine, and the third targeting the lumbar spine. For Kaplan–Meier analyses an event was counted when mice required euthanasia due to tumor-related morbidity. Mice requiring euthanasia for non-tumor-related reasons (weight loss, physical trauma) were censored. Animal experiments were approved by the Animal Ethics Committee of the Telethon Kids Institute and performed in accordance with Australia’s Code for the Care and Use of Animals for Scientific Purposes.

### Immunohistochemistry

BALB/c^*nu/nu*^ mice (Animal Resources Centre) bearing intracranial D425GiL xenografts were treated with either vehicle (20% Captisol^®^), veliparib (two 12.5 mg/kg doses, p.o., 8 h apart), 2 Gy radiotherapy alone (delivered using the XRAD SmART), or a combination of veliparib and 2 Gy radiotherapy (*n* = 3–4 per group). Mice were anesthetized after 24 h, perfused with PBS followed by 4% paraformaldehyde (PFA) in PBS and brains were further fixed in 4% PFA/PBS overnight at 4°C prior to paraffin embedding. Tissue sections (5 μm) underwent antigen retrieval in citrate buffer before immunostaining with the following primary antibodies: cleaved caspase-3 (BD, #559565, 1:500), γH2AX (CST, #9718S, 1:500), phospho-histone H3^*Thr*3^ (CST, #9714, 1:100), PARP-1 (Abcam, #32138, 1:500). Sections were developed using an Elite ABC kit and NovaRED substrate, then counterstained with Gill’s hematoxylin (Vector Laboratories). Positively stained cells were quantified using a Nuance spectral unmixing camera and InForm software (Perkin Elmer).

### Statistical Analysis

Prism v8.1.2 was used to analyze results. Comet assays were compared using unpaired two-tailed Student’s *t*-tests for each timepoint, using Holm-Sidak correction for multiple comparisons. Comparison of treatments in colony forming assays, immunofluorescence and immunohistochemistry was performed using a one-way ANOVA with Holm-Sidak multiple testing correction. Comparison of Kaplan–Meier survival curves was performed using the Mantel–Cox test. Where multiple testing correction was carried out, adjusted *p*-values are reported.

## Results

### PARP Inhibition Alone Does Not Reduce Medulloblastoma Cell Viability

To determine the effect of PARP inhibition on medulloblastoma cell viability *in vitro* drug sensitivity assays were performed using veliparib (Supplementary Figures 1A,B). Minimal effect on D425GiL and D283Luc2 medulloblastoma cell viability was observed over a large range of concentrations. The ED50 was estimated to be >25 μM.

### Inhibition of PARP Delays Repair of Radiation-Induced DNA Damage

**FIGURE 1:**
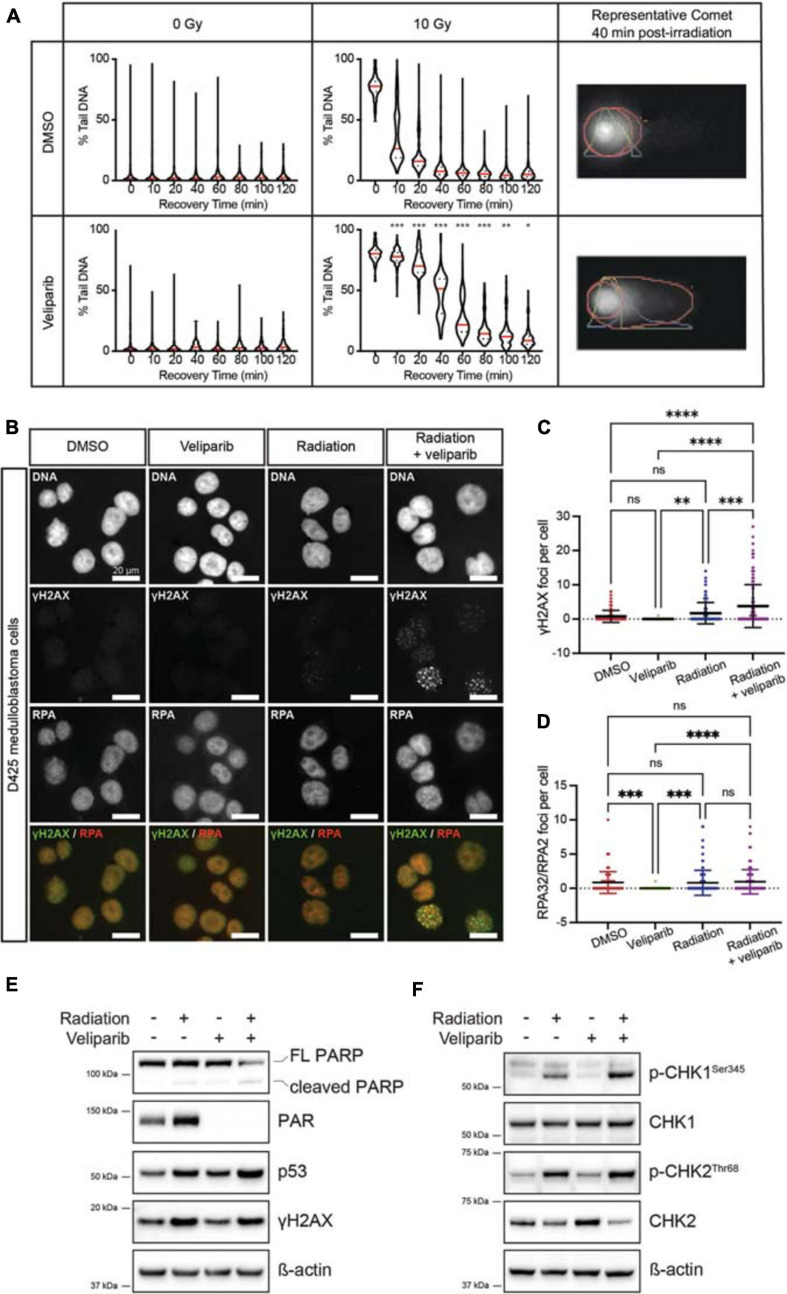
PARP inhibition reduces the repair rate of radiation-induced DNA damage. **(A)** Comet assays were performed to detect DNA damage post-irradiation. Violin plots showing DNA damage (measured as the percent of DNA in the comet tail) for D425GiL medulloblastoma cells treated with DMSO or veliparib; and exposed to 0 or 10 Gy γ-radiation. Mean values for each timepoint are shown in red. *p < 0.05, **p < 0.01, ***p < 0.001. Shown are representative images of comets with the head and tail regions outlined in red for each treatment at 40 min recovery time post-irradiation. **(B)** Representative fluorescent microscopy images of D425 medulloblastoma cells stained for DNA damage foci (γH2AX, green) and DNA repair foci (RPA32/RPA2, red), shown as single channel and composite images. Scale bar represents 20 μm. **(C,D)** Quantification of γH2AX foci **(C)** or RPA32/RPA2 **(D)** foci per cell. Each point is an individual cell and mean ± SD is shown. **p < 0.01, ***p < 0.001, ****p < 0.0001, ns indicates not significant. **(E,F)** Immunoblots for the indicated proteins in D425GiL medulloblastoma cells treated with DMSO (–) or veliparib and exposed to 0 or 10 Gy γ-radiation. Blots are representative of two independent experiments.

The alkaline comet assay was used to determine the effect of veliparib on radiation-induced DNA damage repair in D425GiL medulloblastoma cells. Veliparib did not induce DNA damage when used as a single agent (Figure 1A). Radiation-induced DNA strand breaks, detected as an increase in the percentage of DNA in the comet tail, were almost completely resolved within 2 h in cells treated with DMSO (Figure 1A). When the cells were exposed to veliparib in combination with radiation, a significant delay in DNA damage repair following irradiation was observed (Figure 1A). These results indicate that PARP inhibition reduces the repair rate of radiation-induced DNA damage in human medulloblastoma cells.

### PARP Inhibition Increases DNA Damage Foci

The effect of veliparib, radiation or combination treatment on the number of γH2AX foci (indicating DNA damage) and RPA32/RPA2 foci (indicating DNA repair) in medulloblastoma cells was determined (Figure 1B). In D425 cells treated with DMSO or veliparib few γH2AX foci were present but foci were significantly increased upon irradiation (2 Gy) and then further increased upon co-treatment with veliparib and irradiation (Figures 1B,C). Cells treated with veliparib showed significantly fewer RPA32/RPA2 DNA repair foci compared to DMSO controls, however there were no significant differences in DNA repair foci between cells treated with 2 Gy irradiation alone and combination veliparib/irradiation at the timepoint examined (24 h, Figure 1D). These results were validated in a second medulloblastoma cell line. D283Luc2 cells treated with DMSO or veliparib had few γH2AX foci (Supplementary Figure 2A). Irradiation significantly increased γH2AX foci compared to DMSO controls, and cells treated with combination veliparib/irradiation had significantly increased γH2AX foci compared to irradiation alone (Supplementary Figure 2B). Furthermore, in these cells RPA32/RPA2 foci were also increased in combination veliparib/radiation-treated cells compared to irradiation alone (Supplementary Figure 2C). These data are consistent with the comet assays and suggest that PARP inhibition delays the ability of medulloblastoma cells to repair radiation-induced DNA damage.

### DNA Damage Pathways Are Upregulated Following PARP Inhibition

The DDR following veliparib and/or radiation exposure was further assessed using immunoblotting (Figures 1E–F). In D425 cells treated with veliparib, either alone or in combination with irradiation, clear inhibition of PARP was observed as a reduction in PAR protein. As expected, DNA damage (as measured by γH2AX abundance) was increased in irradiated medulloblastoma cells, either alone or in combination with veliparib. Notably, although p53 abundance was increased in cells treated with irradiation alone, it was further increased in combination-treated cells indicating exacerbated cellular stress. Radiation induces cell cycle arrest via activation of ATM, ATR and phosphorylation of the downstream regulators CHK1 and CHK2 (Huang and Zhou, 2020), which become dephosphorylated upon the completion of DNA repair. As expected, radiation induced both CHK1 and CHK2 phosphorylation, and this was increased by co-treatment with veliparib. Similar effects were observed in D283Luc2 medulloblastoma cells (Supplementary Figure 2D). Since CHK1 and CHK2 phosphorylation were increased following combination exposure of cells to veliparib and radiation we also investigated the effects of treatment on cell cycle progression using flow cytometry. D425 and D283 medulloblastoma cells were treated with DMSO, veliparib, radiation or both veliparib and radiation and cell cycle progression was assessed. Radiation induced a reduction in DNA synthesis and robust G2 arrest in both cell lines; however, no difference in cell cycle arrest or recovery was observed in the presence of veliparib (Supplementary Figure 3). Overall, the immunoblotting results indicate persistent activation of the DDR pathway when veliparib is combined with radiation and support our data suggesting veliparib delays DNA repair in medulloblastoma cells. Immunoblotting data also revealed that, in both cell lines, PARP cleavage was increased following combination treatment suggesting that apoptosis is induced.

### PARP Inhibition Radiosensitizes Medulloblastoma Cells *in vitro*

**FIGURE 2:**
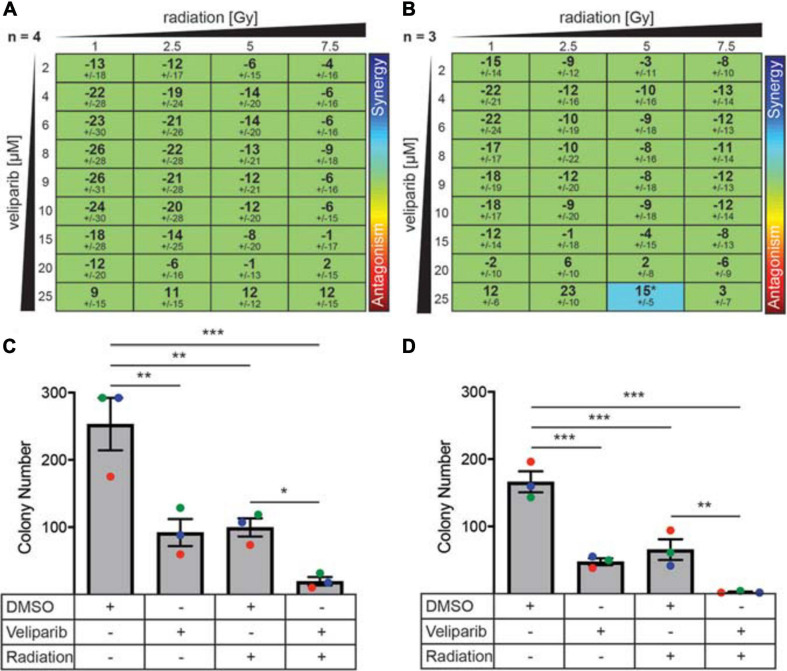
PARP inhibitors enhance radiation-induced cytotoxicity and decrease colony forming ability in medulloblastoma cells. **(A)** D425GiL and **(B)** D283Luc2 cells were treated with increasing doses of radiation combined with veliparib and the mean ± SD synergy score for each combination was calculated using the Loewe Additivity model. Values greater than or less than zero indicate synergy or antagonism, respectively (indicated by the rainbow heat map). The number (*n*) of independent experiments used in the analysis is shown, and statistical comparisons were performed as described by Di Veroli et al., 2016. *p < 0.05. **(C)** D425Gil and **(D)** D283Luc2 cells were treated with DMSO, veliparib, 2 Gy irradiation or a combination of veliparib and irradiation as indicated and plated in methylcellulose. The mean number of colonies formed ± SEM is shown from three independent experiments (indicated in different colors). Groups were compared using one-way ANOVA with Holm–Sidak multiple testing correction. *p < 0.05, **p < 0.01, ***p < 0.001.

Due to its effect on DNA damage repair, we investigated if there was a synergistic effect of combining veliparib and radiotherapy *in vitro* using three different mathematical models of measuring drug-radiation interactions (Bliss, 1939; Loewe, 1953; Tan et al., 2012): the Loewe Additivity method (Figures 2A,B), Bliss Independence model, and the highest single agent (HSA) model (both shown in Supplementary Figure 4). In D425Gil cells, veliparib and radiation was neither synergistic nor antagonistic across multiple different doses indicating an additive interaction (Figure 2A) and in D283Luc2 cells, most experimental conditions were additive, although the combination of 25 μM veliparib and 5 Gy radiation demonstrated significant synergy (Figure 2B). These results show that PARP inhibition in combination with radiotherapy reduces medulloblastoma cell viability *in vitro*.

The limitation of these interaction assays is that they are performed over a short time frame (72 h). To further investigate the ability of veliparib and radiation to impact medulloblastoma proliferative capacity, clonogenicity assays were performed (Figures 2C,D). Medulloblastoma cells treated with radiation (2 Gy) showed significantly reduced colony forming capacity compared to controls (D425, *p* < 0.01; D283, *p* < 0.001). Veliparib alone also significantly impaired colony forming capacity (D425Gil, *p* < 0.01; D283Luc2, *p* < 0.001), which was in contrast to the failure of veliparib to measurably reduce cell viability in the dose response assays. Of note, the combination of veliparib with radiation resulted in a further significant reduction in colony number compared to radiation alone (D425Gil, *p* < 0.05; D283Luc2, *p* < 0.01). These results indicate that veliparib has a radiosensitizing effect on medulloblastoma cells and impairs colony forming capacity.

### Veliparib in Combination With Radiation Increases Medulloblastoma Cell Apoptosis *in vivo*

**FIGURE 3:**
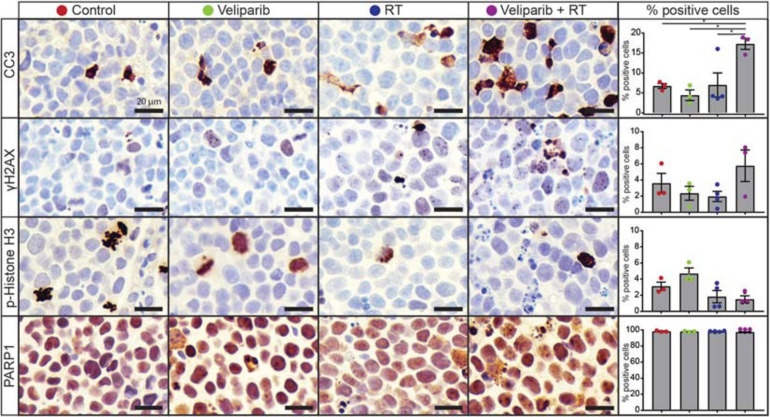
Veliparib in combination with radiotherapy increases apoptosis in an orthotopic xenograft model of medulloblastoma. Representative images are shown of immunohistochemistry for cleaved caspase 3 (CC3), γH2AX, phospho(p)-histone H3, and PARP1 from D425GiL xenografts in mice treated with vehicle, veliparib, radiotherapy (RT) or the combination of both veliparib and RT. The percentage of positively stained medulloblastoma cells for each antibody were quantified from three independent fields of view per tumor from *n* = 3 or 4 mice per treatment group. Error bars represent SEM. Scale bar represents 20 μm. *p < 0.05.

Given the effects of veliparib and radiotherapy co-treatment on medulloblastoma cells *in vitro*, we examined the effect of treatment on medulloblastomas *in vivo* by immunohistochemical analysis of orthotopic D425GiL xenografts grown in immune-deficient mice (Figure 3). Combined treatment of mice with veliparib and radiotherapy significantly increased the proportion of apoptotic medulloblastoma cells (marked by cleaved caspase 3) compared to controls or single agents. There was a trend toward increased DNA damage and decreased mitosis (marked using γH2AX and phospho-histone H3, respectively) in medulloblastomas treated with combination therapy, but these results were not statistically significant at the timepoint examined. Notably, no difference in PARP1 staining was observed in the tumors of veliparib-treated mice, likely due to the 24-h timepoint examined, at which point very little veliparib remains in the brain (Gupta et al., 2016).

### Veliparib in Combination With Radiotherapy Increases Animal Survival

**FIGURE 4:**
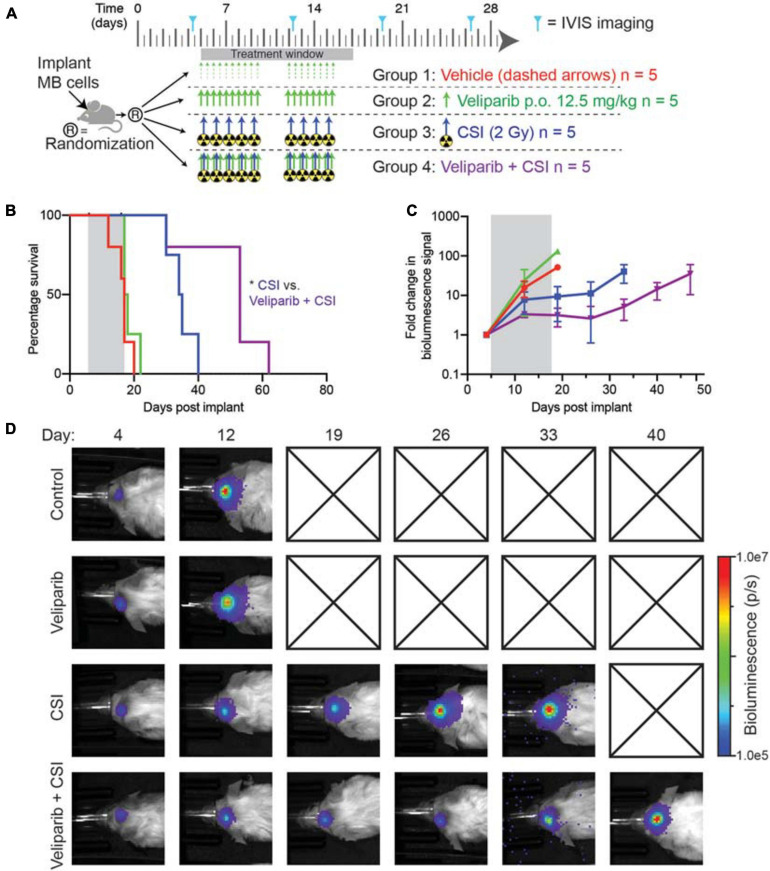
Combination veliparib and CSI extends survival of mice with medulloblastoma. **(A)** Preclinical mouse treatment protocol for mice with orthotopic D425GiL Group 3 medulloblastoma for data shown in **(B-D)**, *n* = 5 mice per group. **(B)** Survival curves for mice treated as indicated in **(A)**. Mantel–Cox tests compared the combination-treated group with CSI alone: *p < 0.05. **(C)** Bioluminescence measurements from the animals shown in **(B)**. Data are represented as the fold change in mean ± SD bioluminescence flux measured over time with a representative mouse from each group shown in **(D)**. Checked boxes indicate that the animal was euthanized prior to the timepoint shown.

Given the increased apoptosis observed following combination veliparib/radiotherapy treatment, the effect of veliparib in combination with CSI on medulloblastoma growth and overall animal survival was assessed. Mice with D425GiL medulloblastomas were treated as shown in Figure 4A and monitored until the development of tumor-related morbidity (Figure 4B). Control mice had a median tumor-free survival of 17 days. Veliparib treatment alone had no impact on animal survival, with a median survival of 17.5 days. As expected, radiotherapy (CSI) increased median survival compared to control mice (34.5 versus 17 days, respectively). Combination treatment with veliparib and radiotherapy significantly increased survival compared to radiotherapy alone, with a median survival of 53 days (*p* < 0.05, Figure 4B). Two mice were censored during treatment, one in the veliparib alone group (diarrhea) and one in the radiotherapy alone group (anesthesia-associated death). In concordance with the survival data, bioluminescence flux in mice treated with vehicle or veliparib was similar, while CSI delayed tumor growth (Figures 4C,D). Tumor growth was further delayed in mice treated with veliparib in combination with CSI; however, tumor regression was not observed.

## Discussion

This study aimed to identify treatments to improve medulloblastoma patient outcomes. We tested inhibition of the DDR pathway in combination with radiotherapy in *MYC-*amplified Group 3 medulloblastoma. Veliparib, a small molecule inhibitor of PARP1/2, was assessed for its ability to inhibit DNA repair in two human medulloblastoma cell lines.

*In vitro* assays were performed to test the effect of veliparib on medulloblastoma cell viability. Doses of up to 25 μM had no effect on the viability of medulloblastoma cells over a short time course. Higher doses were not tested, as they are unlikely to be achieved *in vivo* in the brain or brain tumor. Recent studies have shown peak concentrations of 0.71 and 3.0 μmol/L were achieved in mouse brain and brain tumors respectively, using the same veliparib dosing schedule as this study (Donawho et al., 2007; Gupta et al., 2016). It is important to note that since these medulloblastoma cell lines are not deficient in HR, we did not expect to see synthetic lethality, and therefore did not expect veliparib alone to affect viability; although, veliparib exposure did inhibit the colony-forming ability of medulloblastoma cells over a longer experimental period *in vitro*. Despite showing minimal cytotoxicity using the metabolic reagent alamarBlue, veliparib significantly reduced medulloblastoma cell colony forming capacity to a similar extent as radiation. To test the potential of veliparib to act as a radiosensitizing agent, we examined the interaction between veliparib and radiotherapy using both alamarBlue and clonogenicity assays. The combination of veliparib and radiation was found to be mostly additive with some synergy, suggesting that it may be an efficient radiosensitizer in medulloblastoma. Notably, the combination of veliparib and radiation significantly reduced colony formation for both cell lines. This demonstrates the limitation of short-term metabolic assays in understanding the impact of drug treatment *in vitro*, and adds evidence to suggest that veliparib can enhance medulloblastoma control in combination with radiotherapy. This potential radiosensitizing ability of PARP inhibition is consistent with observations made with veliparib in other brain cancers such as glioblastoma (Jue et al., 2017), and the PARP inhibitor olaparib on D283 medulloblastoma cells (Van Vuurden et al., 2011).

Mechanistically, comet assays demonstrated that veliparib was able to delay repair of radiation-induced DNA damage in the medulloblastoma cells tested. This is consistent with PARP1 deficient cells, which also exhibit delayed but not ablated DDR activation (Haince et al., 2007). However, veliparib treated cells had not completely repaired the radiation-induced DNA damage by the end of the experiment, warranting further investigation on the lasting mutational burden in these cells. This may be of particular importance since pediatric brain tumors are known to have low mutational burden, partially contributing to the relative lack of success with some immunotherapies (Wang et al., 2019; Patel et al., 2020). In further support of a potential therapeutic benefit that may be achieved by combining veliparib and radiation is the increased γH2AX foci in medulloblastoma cells following combination treatment compared to radiation alone. Additionally, in the *TP53* mutant D425 cell line, veliparib markedly reduced the formation of DNA repair foci, and when combined with irradiation, no increase in DNA repair foci was seen in veliparib-treated cells despite increased DNA damage. This effect was not seen in the *TP53* wild-type D283Luc2 cells, suggesting that p53 deficient cells may show an increased response to PARP inhibition in combination with radiotherapy. In combination with radiation, veliparib also increased downstream activation of the DDR pathway, including increases in p53, phosphorylated CHK1^*Ser*345^ and phosphorylated CHK2^*Thr*68^, suggesting the persistence of DNA damage in the combination treated cells.

Following these encouraging *in vitro* results, veliparib was tested as a radiosensitizing agent in a mouse model of medulloblastoma. Immunohistochemistry demonstrated that a single dose of veliparib in combination with radiotherapy significantly increased intratumoral apoptosis, suggesting that veliparib improves radiation-induced medulloblastoma cell death *in vivo*. A trend toward increased DNA damage and decreased mitosis was observed in combination treatment groups, although this was limited by the small number of animals examined and was not statistically significant. PARP-1 expression was high in D425Gil tumors, consistent with a previous study showing high expression in D283 medulloblastoma cells and clinical samples (Van Vuurden et al., 2011), although this expression remained high in groups treated with veliparib. Veliparib has been shown to cross the blood–brain barrier (Donawho et al., 2007) and our observed increase in apoptosis confirms intratumoral drug penetration in this model. However, pharmacokinetic studies in brain tissue show veliparib concentrations peak shortly after administration but are almost absent 6 h thereafter (Gupta et al., 2016), likely explaining why no change in PARP-1 expression was observed in our samples examined 24 h post-treatment. Due to the short half-life of veliparib, we chose twice daily administration to assess the impact of treatment on animal survival, similar to Jue et al. (2017).

The potential of veliparib to extend survival was examined *in vivo* using an orthotopic xenograft mouse model of Group 3 medulloblastoma. Veliparib had no effect on survival compared to controls, further confirming the *in vitro* findings that veliparib monotherapy was insufficient to induce medulloblastoma cell death. CSI alone increased survival over control groups but did not cure mice, consistent with clinical outcomes. The addition of veliparib to CSI significantly increased survival beyond that of radiotherapy alone, confirming our *in vitro* and immunohistochemical findings, and demonstrating the radiosensitizing potential of veliparib in an orthotopic xenograft model of medulloblastoma for the first time.

While veliparib was studied due to its clinical advancement at the time, many other PARP inhibitors with greater PARP trapping abilities and more favorable pharmacokinetics have since been investigated (Pommier et al., 2016). Several adult clinical trials have examined, or are in the process of examining the safety of PARP inhibitors such as olaparib (NCT03212742), rucaparib (NCT03542175), and niraparib (NCT03076203) in combination with radiotherapy. Other PARP inhibitors have also moved into clinical trial for pediatric brain cancers. Olaparib has been tested as a single agent (Pediatric MATCH, NCT03233204) (Takagi et al., 2019) in pediatric solid tumors, and was well tolerated. One patient with pediatric high-grade glioma who was treated with olaparib and temozolomide demonstrated a 2 year durable response (Valiakhmetova et al., 2020). Talazoparib has also been tested with temozolomide in pediatric solid tumors, though limited anti-tumor activity was observed in CNS tumors (Schafer et al., 2020), likely due to poor blood-brain barrier penetrance (Kizilbash et al., 2017).

Collectively our preclinical data demonstrate that PARP inhibition can improve animal survival in combination with radiotherapy. Although veliparib may not be the optimal PARP inhibitor to take forward clinically, we provide evidence that radiosensitization through PARP inhibition shows promise for improving the efficacy of radiotherapy in medulloblastoma.

## Data Availability Statement

The raw data supporting the conclusions of this article will be made available by the authors, without undue reservation.

## Ethics Statement

The animal study was reviewed and approved by the Telethon Kids Institute Animal Ethics Committee.

## Author Contributions

RE, ME, KM, and NG designed the study. JBu, PD, HH, BC, MK, JBy, JW, and RE performed the experiments. JBu, PD, MH, and RE analyzed the data. JBu, PD, MH, NG, and RE prepared and intellectually assessed the manuscript. All authors read and approved the final version of the manuscript.

## Conflict of Interest

KM was employed by company Brain Cancer Consultancy. The remaining authors declare that the research was conducted in the absence of any commercial or financial relationships that could be construed as a potential conflict of interest.
