# Supplementary material for: Veliparib Is an Effective Radiosensitizing Agent in a Preclinical Model of Medulloblastoma
Source: Front Mol Biosci. 2021 Apr 29;8:633344. doi: 10.3389/fmolb.2021.633344 (PMC8116896; doi:10.3389/fmolb.2021.633344)
Supplement: Supplementary file 1 [file Table_1.DOCX]

Supplementary Material

# Supplementary Methods

1.1 Irradiation methods

Irradiations performed with the X-RAD SmART system used a 225 kVp beam (0.32 mm Cu filtration, 1.0 mm Cu HVL) with a large source size at 13 mA tube current, delivering approximate dose-rates of between 2.8 Gy/min and 3.5 Gy/min at isocentre (depending on collimator size). The X-ray source is also used for image-based localisation, including planar X-ray images and cone-beam computed tomography (CBCT) images. The X-RAD device and SmART-Plan system are maintained via a routine quality assurance program (Feddersen et al., 2019). The spatial and dosimetric accuracy of the system has previously been established (van Hoof et al., 2013;Lindsay et al., 2014;Feddersen et al., 2019). Determination of device output for absorbed dose calculations is made according to the protocol of the American Association of Physicists in Medicine using a calibration traceable to a primary standards laboratory (Ma et al., 2001). Dose calculation was undertaken using the EGSnrc Monte Carlo code based on medium information derived from CBCT images (Kawrakow, 2000). Doses are reported as dose-to-medium. For irradiation of 384 well plates, the dose plan was designed to achieve at least 90% of wells receiving the prescribed doses of 1, 2.5, 5 or 7.5 Gy. For craniospinal irradiation in mice, the dose plan was designed to achieve coverage of the central nervous system with at least 90% of the brain receiving 1.8 Gy per fraction for each 2 Gy dose.

Irradiations performed using the ^137^caesium source irradiator provided a dose-rate of 3.87 Gy/min, calibrated with a National Institute of Standards and Technology (NIST) traceable standard.

# Supplementary Figures.

#
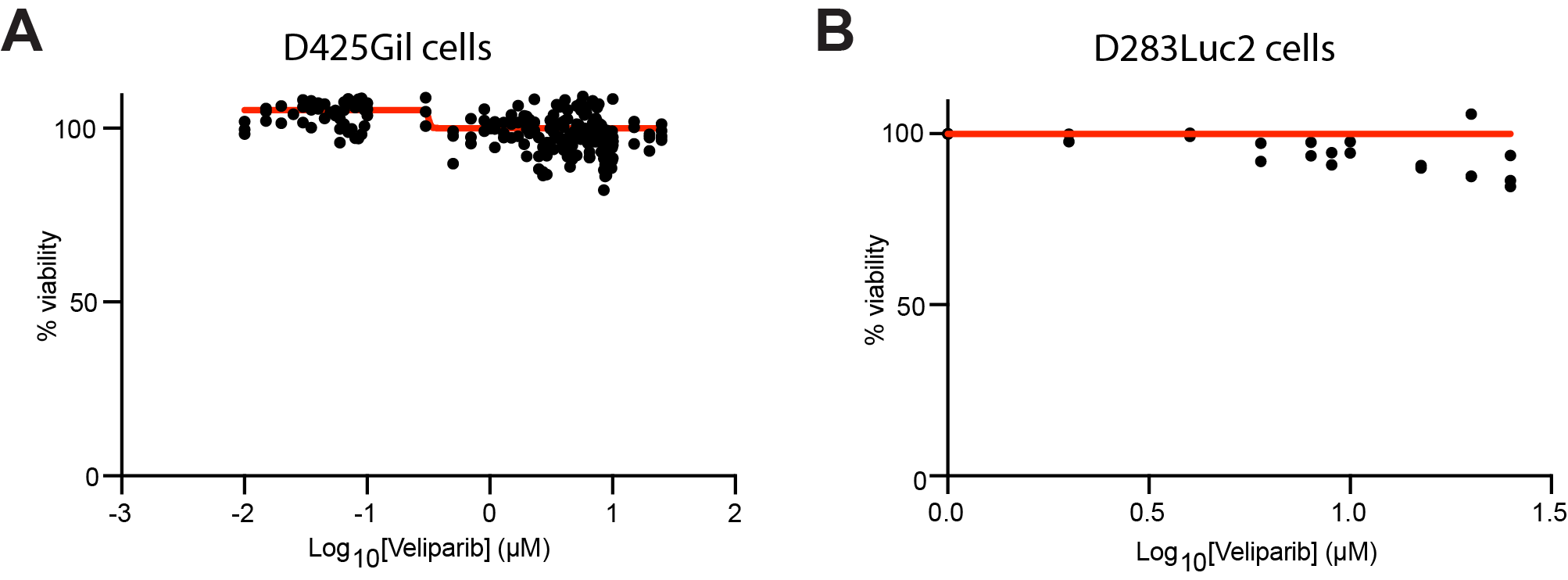


# Supplementary Figure S1. Veliparib interacts with radiotherapy in an additive manner in medulloblastoma cells. (A-B) Dose response curves for veliparib in (A) D425Gil and (B) D283Luc2 cells. A minimum of three experimental replicates were used to calculate the response curves (red) using GraphPad Prism software.


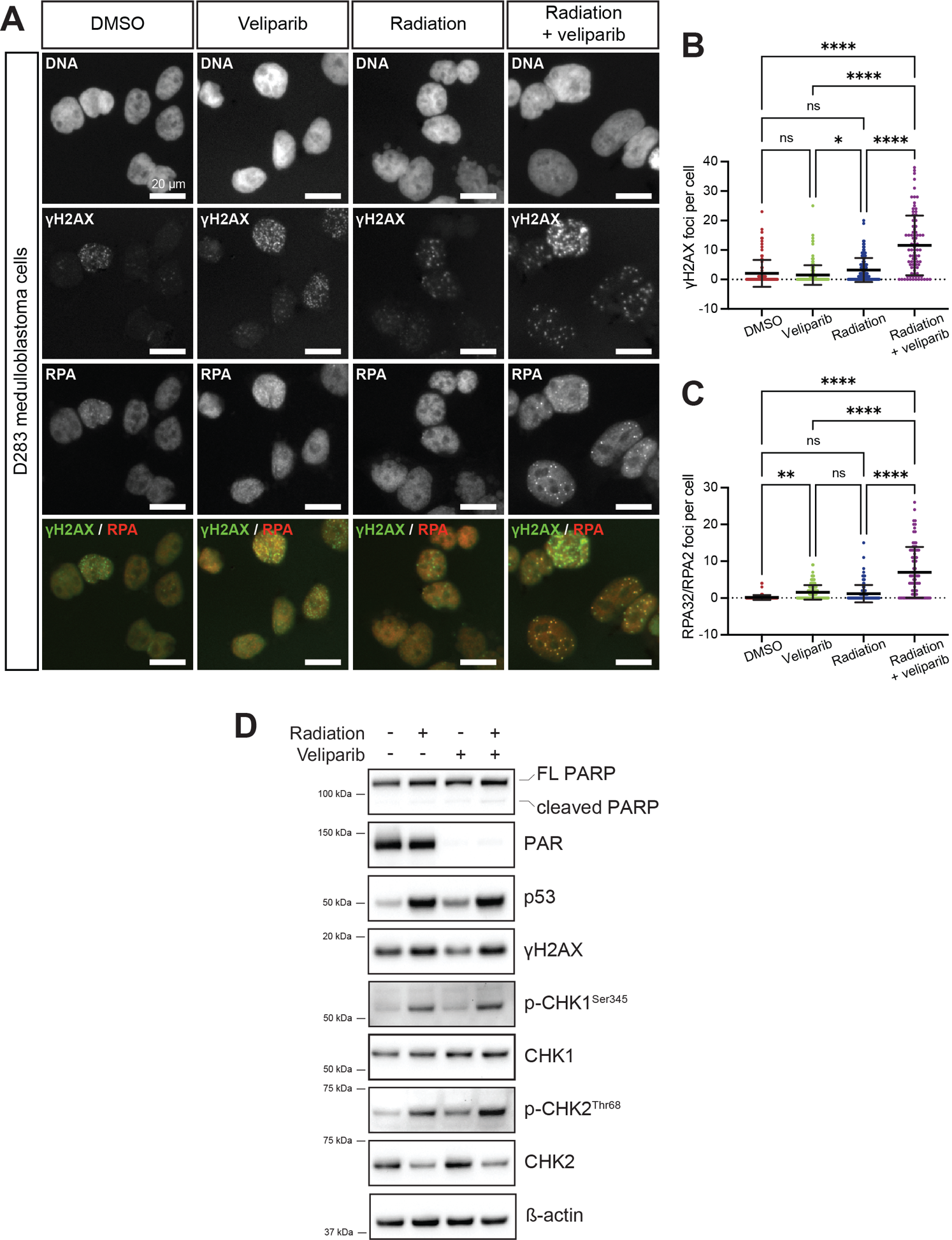


**Supplementary Figure S2.** Effects on DNA damage and repair of veliparib in combination with irradiation in D283Luc2 medulloblastoma cells. A) Representative immunofluorescent images of D283Luc2 cells stained for DNA damage foci (γH2AX, green) and DNA repair foci (RPA32/RPA2, red), shown as single channel and composite images. Scale bar represents 20 μm. B) Quantification of γH2AX DNA damage foci per cell. C) Quantification of RPA32/RPA2 DNA repair foci per cell. Data is shown as mean ± standard deviation. *p < 0.05, **p < 0.01, ****p < 0.0001, ns = not significant. D) Immunoblots showing expression of the indicated proteins in D283Luc2 medulloblastoma cells treated with DMSO or veliparib; and exposed to 0 or 10 Gy γ-radiation. Blots are representative of two independent experiments.

**
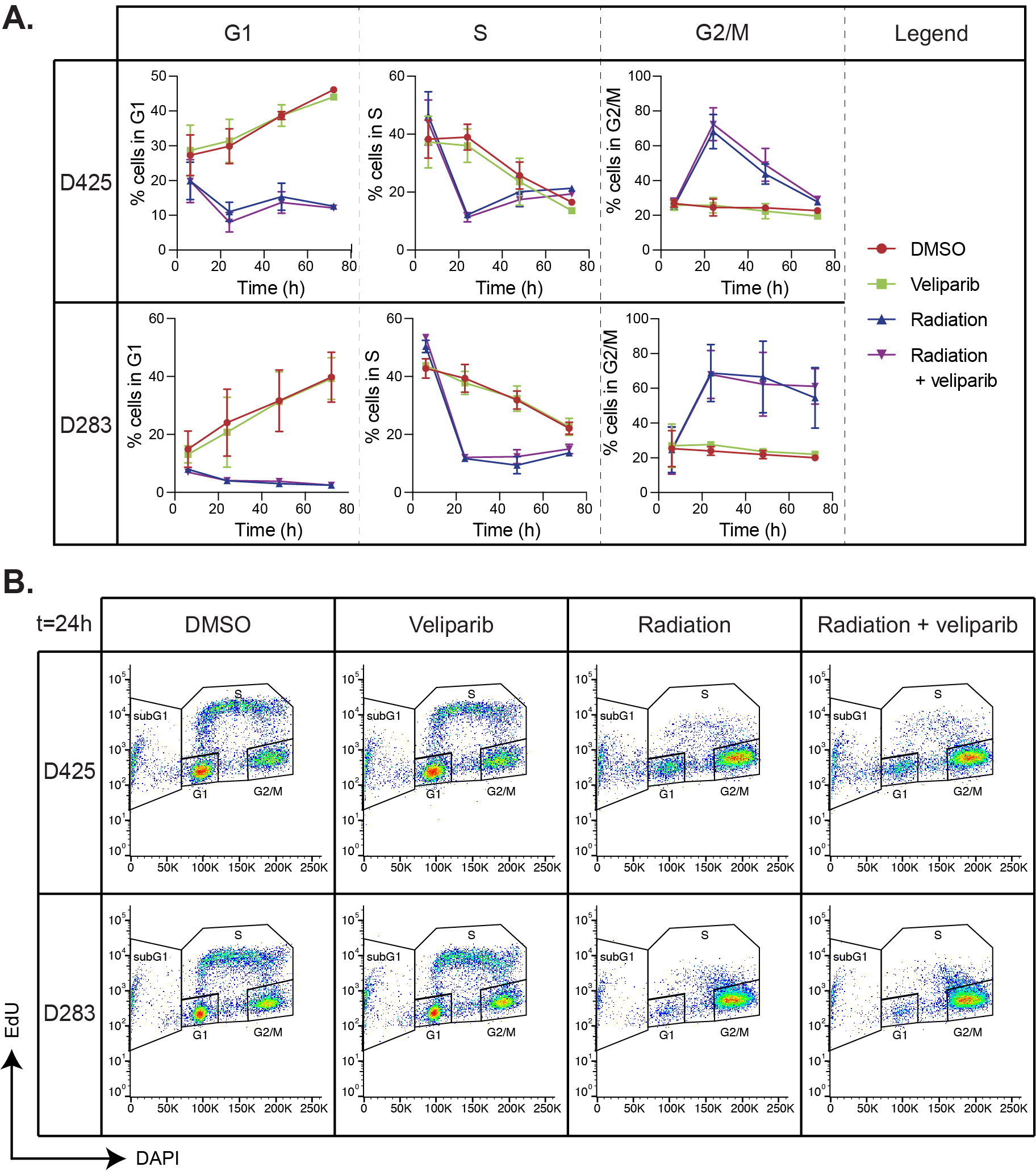
**

**Supplementary Figure S3.** The addition of veliparib to irradiation does not affect cell cycle progression of medulloblastoma cells. (A) The percentage of D425 or D283 medulloblastoma cells, treated as indicated, in G1, S or G2/M phase of the cell cycle was determined over time (x-axis) using flow cytometry. Data is shown as mean ± standard deviation. (B) Representative dot plots for EdU (to mark DNA synthesis) and DAPI (to measure DNA content) are shown from D425 or D283 medulloblastoma cells 24 h post-treatment.


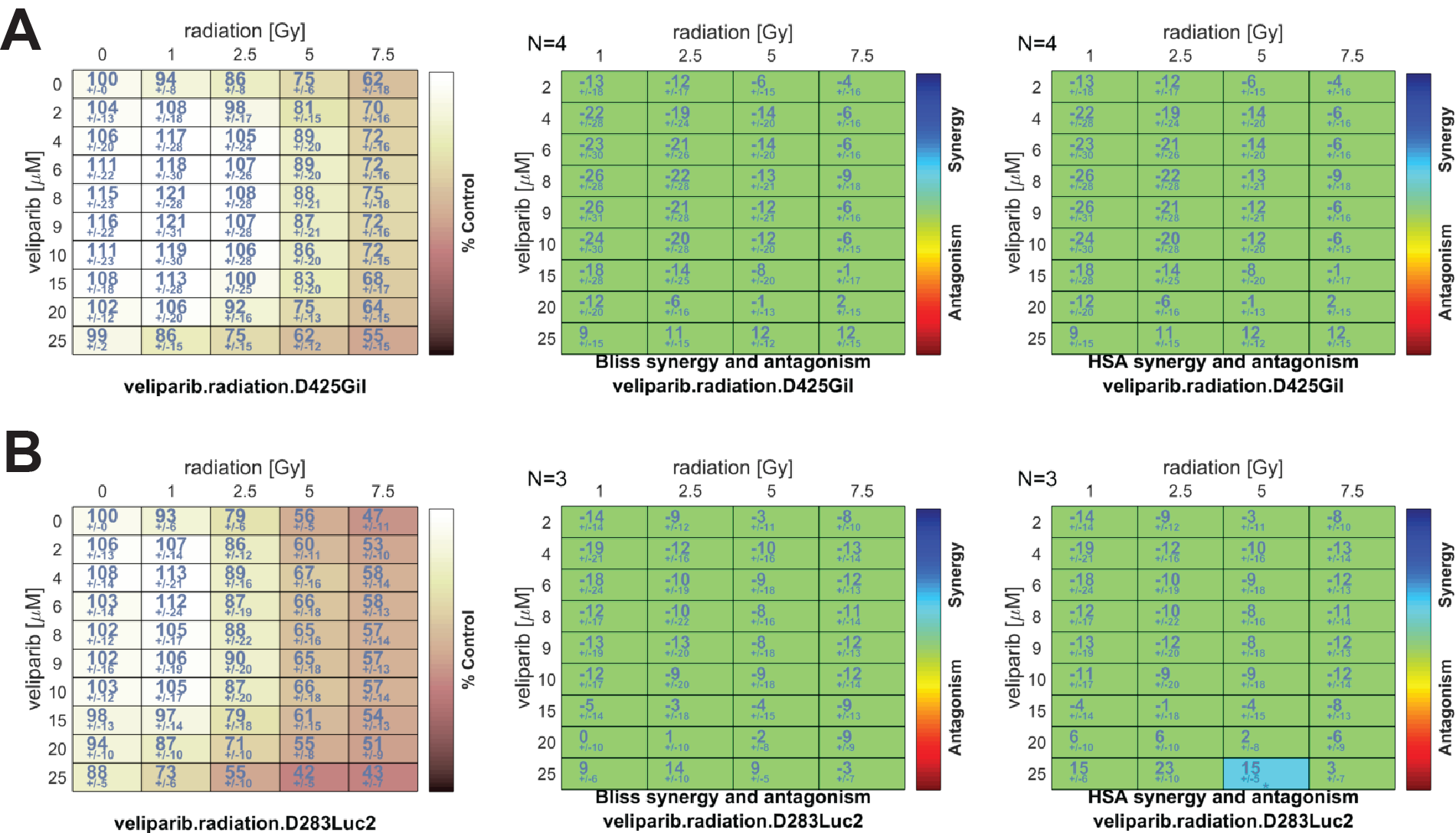


# Supplementary Figure S4. Combinatorial effects of PARP inhibition in combination with radiation in medulloblastoma cells. Increasing concentrations of veliparib were applied to (A) D425Gil or (B) D283Luc2 cells as indicated before cells were exposed to increasing amounts of radiation. Viability of cells after 72 hours is shown (top left, dose response matrix); and the observed drug-radiation interactions were compared to predicted data modelled using the Bliss and HSA algorithms as labelled. Synergistic, additive or antagonistic observations are indicated by the rainbow-colored heat map.

**3 Supplementary References**

Feddersen, T.V., Rowshanfarzad, P., Abel, T.N., and Ebert, M.A. (2019). Commissioning and performance characteristics of a pre-clinical image-guided radiotherapy system. *Australasian Physical & Engineering Sciences in Medicine* 42**,** 541-551.

Kawrakow, I. (2000). Accurate condensed history Monte Carlo simulation of electron transport. I. EGSnrc, the new EGS4 version. *Medical Physics* 27**,** 485-498.

Lindsay, P.E., Granton, P.V., Gasparini, A., Jelveh, S., Clarkson, R., Van Hoof, S., Hermans, J., Kaas, J., Wittkamper, F., Sonke, J.-J., Verhaegen, F., and Jaffray, D.A. (2014). Multi-institutional dosimetric and geometric commissioning of image-guided small animal irradiators. *Medical Physics* 41**,** 031714.

Ma, C.-M., Coffey, C.W., Dewerd, L.A., Liu, C., Nath, R., Seltzer, S.M., and Seuntjens, J.P. (2001). AAPM protocol for 40–300 kV x-ray beam dosimetry in radiotherapy and radiobiology. *Medical Physics* 28**,** 868-893.

Van Hoof, S.J., Granton, P.V., and Verhaegen, F. (2013). Development and validation of a treatment planning system for small animal radiotherapy: SmART-Plan. *Radiotherapy and Oncology* 109**,** 361-366.
